# Supplementary material for: Cold War spy satellite images reveal long-term declines of a philopatric keystone species in response to cropland expansion
Source: Proc Biol Sci. 2020 May 20;287(1927):20192897. doi: 10.1098/rspb.2019.2897 (PMC7287353; doi:10.1098/rspb.2019.2897)
Supplement: Supplementary Material for 'Cold War spy satellite images reveal delayed declines of a philopatric keystone species in response to cropland expansion' [file rspb20192897supp1.pdf]

Supplementary Material

for

Cold War spy satellite images reveal long-term declines of a philopatric keystone species in response to cropland expansion

Catalina Munteanu[1,2,3*], *Johannes Kamp*[4], *Mihai Daniel Nita*[3], *Nadja Klein*[5], *Benjamin M Kraemer*[6], *Daniel Müller*[1,2,7], *Alyona Koshkina*[4,8], *Alexander V. Prishchepov*[9,10], *Tobias Kuemmerle*[1,8]

[1] Geography Department, Humboldt University Berlin, Unter den Linden 6, 10099 Berlin, Germany; [2] Leibniz Institute of Agricultural Development in Transition Economies (IAMO), Theodor Lieser Straße 2, 06120 Halle (Saale), Germany; [3] Department of Forest Engineering, Faculty of Silviculture and Forest Engineering, Transylvania University of Brasov, 1 Sirul Beethoven, Brasov, Romania; [4] Institute of Landscape Ecology, University of Münster, Heisenbergstr. 2, 48149 Münster, Germany; [5] Chair of Statistics, Humboldt University Berlin, Unter den Linden 6, 10099 Berlin, Germany; [6] Leibniz Institute of Freshwater Ecology and Inland Fisheries, Müggelseedamm 310, 12587 Berlin; Germany,[7] Integrative Research Institute for Transformations in Human-Environment Systems, Humboldt-University Berlin, Unter den Linden 6, 10099 Berlin, Germany; [8] Association for the Conservation of Biodiversity of Kazakhstan (ACBK), 18 Beibitshilik Str., office 406, Astana, 010000, Kazakhstan; [9] Department of Geosciences and Natural Resource Management (IGN), University of Copenhagen, Øster Voldgade 10, DK-1350 København K, Denmark; [10] Institute of Steppe of the Ural Branch of the Russian Academy of Sciences, Pionerskaya str.11, Orenburg 460000, Russia. * Corresponding author: Catalina Munteanu, Geography Department, Humboldt University Berlin, Unter den Linden 6, 10099 Berlin, Germany, E-mail: catalina.munteanu@geo.hu-berlin.de

---

Supplementary Material 1: Corona imagery

Corona is one of several spy satellite missions from the Cold War period, when the US government initiated multiple space-borne photography missions for intelligence purposes [1]. The photographs have been gradually declassified since 1996, and are now available via USGS https://earthexplorer.usgs.gov (see Declassified Data tab). Data coverage is global [2], but most imagery was collected from areas of the former Soviet Union. Due to it's experimental nature, data varies in extent, temporal and spatial resolution. However, high resolution imagery (2-10m) is available for multiple parts of the word, and all data previously acquired are freely available for download on the USGS website [3–5].

In this study, we only use stereo-high and stereo-medium Corona imagery (see Additional Criteria Tab on EarthExplorer) with no or low cloud coverage. We assessed the spatial extent of the data and the cloud cover based on freely available thumbnail images and purchased 12 pairs

of stereographic images dated September 1968 and 1969, with an average ground resolution of 2.3m (listed in the table below). The raw data can be downloaded from the EarthExplorer website, by selecting Declass 1 (1996) in the Data Sets tab and entering the Image unique identifier listed below into the Entity ID column of the Additional Criteria Tab.

Corona imagery used in this study.

Image unique identifier	Latitude	Longitude	Acquisition Date
DS1048-1071DF018	50.8700	65.9950	1968/09/23
DS1048-1071DA018	50.8500	65.9870	1968/09/23
DS1048-1071DF019	50.7200	66.0290	1968/09/23
DS1048-1071DF017	51.0400	65.9530	1968/09/23
DS1048-1071DA020	50.5200	66.0710	1968/09/23
DS1048-1071DA017	51.0000	65.9450	1968/09/23
DS1048-1071DF020	50.5600	66.0710	1968/09/23
DS1048-1071DA019	50.6800	66.0290	1968/09/23
DS1048-2166DA037	49.9900	70.5180	1968/09/29
DS1048-2166DF039	49.7200	70.5800	1968/09/29
DS1048-2166DA039	49.6800	70.5840	1968/09/29
DS1048-2166DA036	50.1300	70.4800	1968/09/29
DS1048-2166DF036	50.1700	70.4720	1968/09/29
DS1048-2166DF038	49.8700	70.5380	1968/09/29
DS1048-2166DA038	49.8300	70.5420	1968/09/29
DS1048-2166DF037	50.0200	70.5090	1968/09/29
DS1052-1040DF071	52.9600	65.1850	1969/09/25
DS1052-1040DF070	53.1200	65.1480	1969/09/25
DS1052-1040DA071	52.9900	65.1520	1969/09/25
DS1052-1040DA070	53.1500	65.1140	1969/09/25
DS1052-1040DA072	52.8400	65.1680	1969/09/25
DS1052-1040DA069	53.3000	65.0890	1969/09/25
DS1052-1040DF072	52.8000	65.2140	1969/09/25
DS1052-1040DF069	53.2700	65.1270	1969/09/25

Supplementary Material 2: Variables used in the occurrence and density modelling.

Our models included a total of 11 explanatory variables. The two bioclimatic variables (mean temperature of the coldest quarter and precipitation of the driest quarter) were extracted from the WorldClim Database [6] for the period 1960-2000 and represented an average across the entire area of each plot. The soil data was extracted from the digitized soil atlas of Kazakhstan (1:2500000), and three classes of soil texture were considered: clay and heavy loam, loam and other [7]. The distance to the nearest river was based on waterway network information extracted from Soviet and Kazakhstan Topographic Maps scaled 1:100,000 (dated aprox. 1970s). The distance to the nearest farm building was also based on the Soviet and Kazakhstan Topographic Maps scaled 1:100,000 for the historical time period. We verified the contemporary location of the farms in Google Earth imagery, because many farms were abandoned after the Soviet Union collapse in 1991 [8], and eliminated the farms that were abandoned following the collapse of the Soviet Union. The average slope for each plot, was derived from the Shuttle Radar Topography Mission 90m Digital Elevation Model [9]. The Normalized Vegetation Difference Index (NDVI) values were used as indicators of vegetation productivity and represented averages during the month of May over the time period between 2008 and 2014. Categorical variables on the time period (historical or contemporary), the study zone (central, north or south) and the plot id were derived from our own data collection.

Variables used in modelling burrow occurrence and density

**Variable name**	**Variable description**	**Unit**	**Source**
bio11	Mean Temperature of Coldest Quarter	degrees Celsius	[6] worldclim.org
bio17	Precipitation of Driest Quarter	mm	[6] worldclim.org
d_farm	Distance to nearest farm building	meter	[10,11], Google Earth
d_river	Distance to nearest river	meter	[10,11]
dominant_lu	Dominant land use within plot	Cropland/ grassland/ other	Corona, Google, Bing, ESRI
ndvi_may	Normalized Difference Vegetation Index for emergence month of May	NDVI	free.vgt.vito.be
period	Time period of analyses (historical vs. contemporary)	contemporary / historical	Corona, Google, Bing, ESRI

slope	Average slope within plot	degrees	srtm.csi.cgiar.org
soil	Soil texture for Kazakhstan	3 classes (see text)	[7]
zone	Area of Kazakhstan in which the plots are located: North, Central, South	N/ C/ S	
plot	Plot area with diameter of 1km for which burrow number were counted	Unique identifier	

Supplementary Material 3: Correlation matrix

Correlation matrix for the continuous variables used in the occurrence and density models. None of the variables were strongly correlated (Pearson correlation < 0.6).

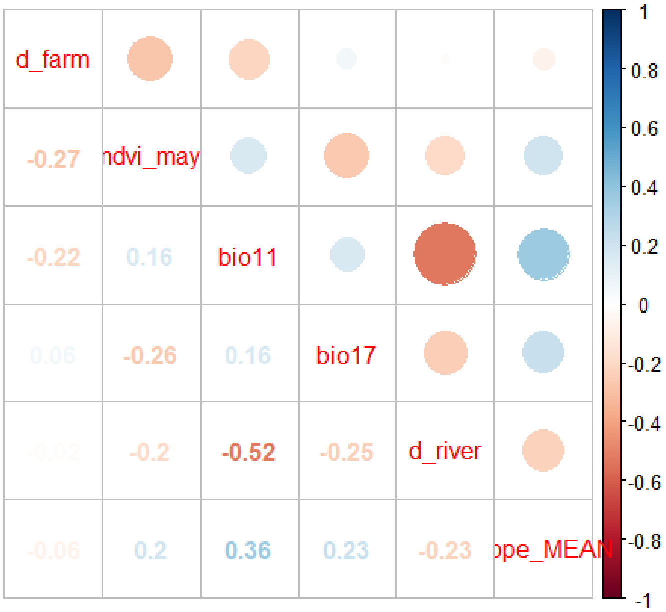

Supplementary Material 4: Model selection

We chose our modelling approach after comparing between a Poisson hurdle model (AIC: 9888.6), a zero inflated Poisson (AIC: 9905.5), a negative binomial hurdle model (AIC: 6373.8), and a zero inflated negative binomial (ZINB, AIC: 6389.5) model for predicting the burrow density. We identified the best fit, given the restrictions imposed by our data and based on assessments of the AIC values, quantile residual plots and rootgrams.

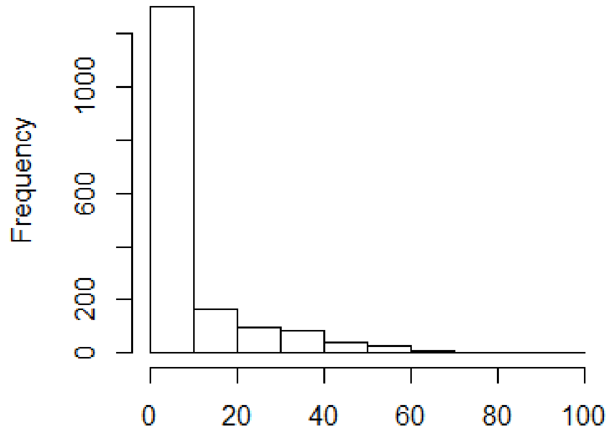

We relied on model diagnostics using QQ-residual plots and rootgrams. We used the QQ-residual plots to check for non-normal patterns and outliers. The rootgrams were used to assess model fit as well as diagnosing issues such as overdispersion and/or excess zeros in count data models. The rootgram depicts the expected values according to the model (red line) in relation to the observed counts (grey bars). The y-axis depicts the square root of the frequencies. Our assessment of the QQplots and rootgrams indicates that the ZINB model and the negative binomial hurdle model fit the data best. We chose to retain the ZINB approach over the hurdle model, because unlike the hurdle model, the ZINB assumes two possible sources of zeros in the data: one from basic environmental unsuitability for marmot presence (binomial part of the model) and the other by chance in areas that are environmentally suitable, but where other processes are at play (count part of the model). In contrast, the hurdle models assume that zeros in the data may only occur due to environmental unsuitability.

Poisson Hurdle Model

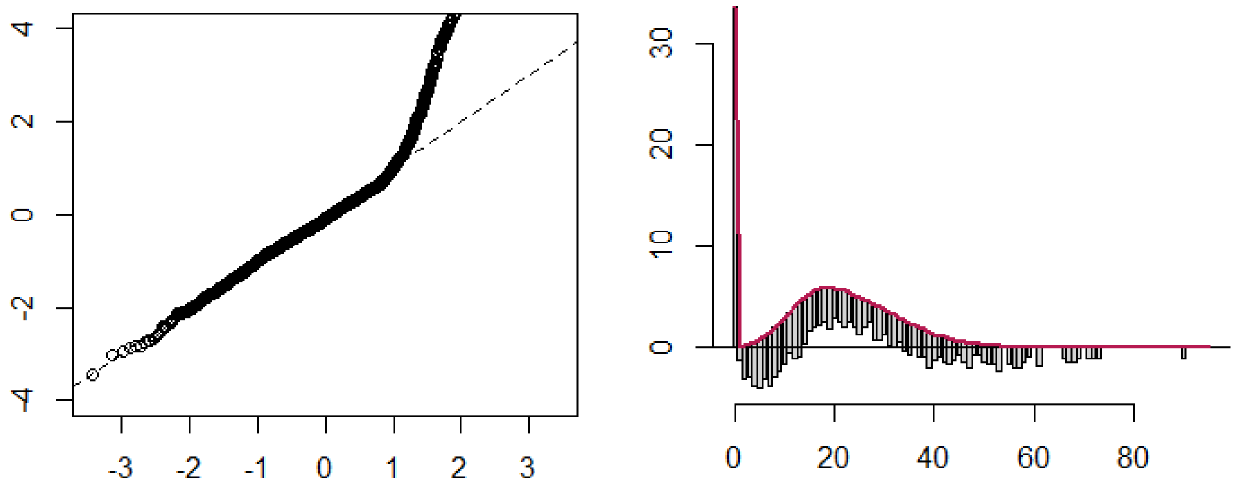

Zero inflated Poisson model

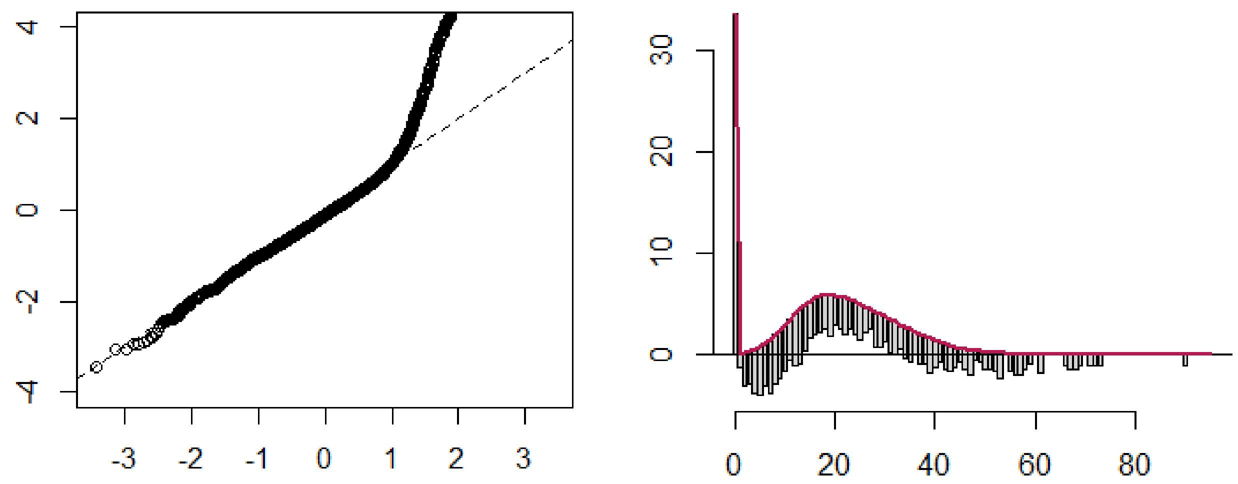

Negative binomial hurdle model

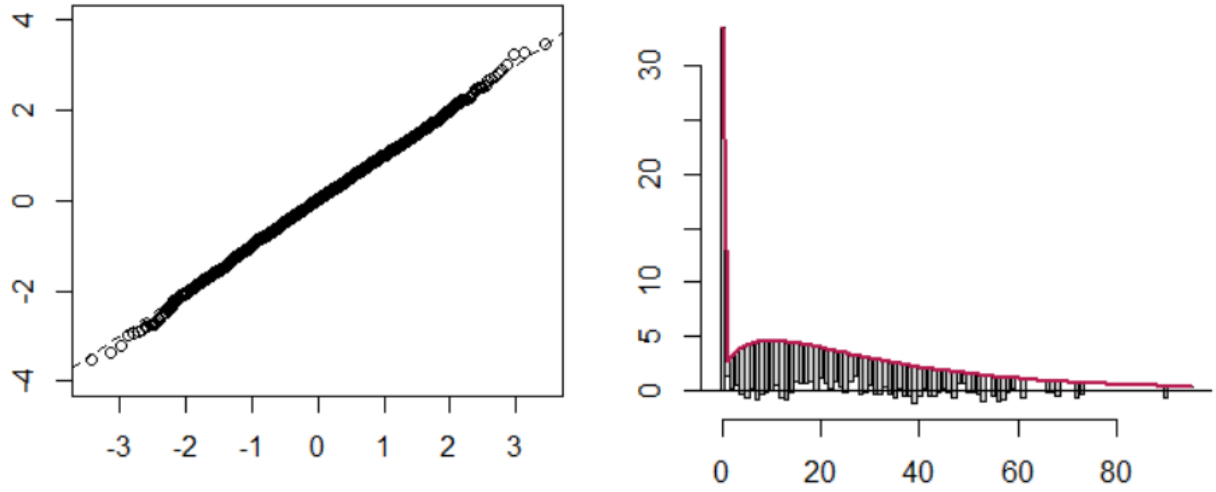

Zero inflated negative binomial model

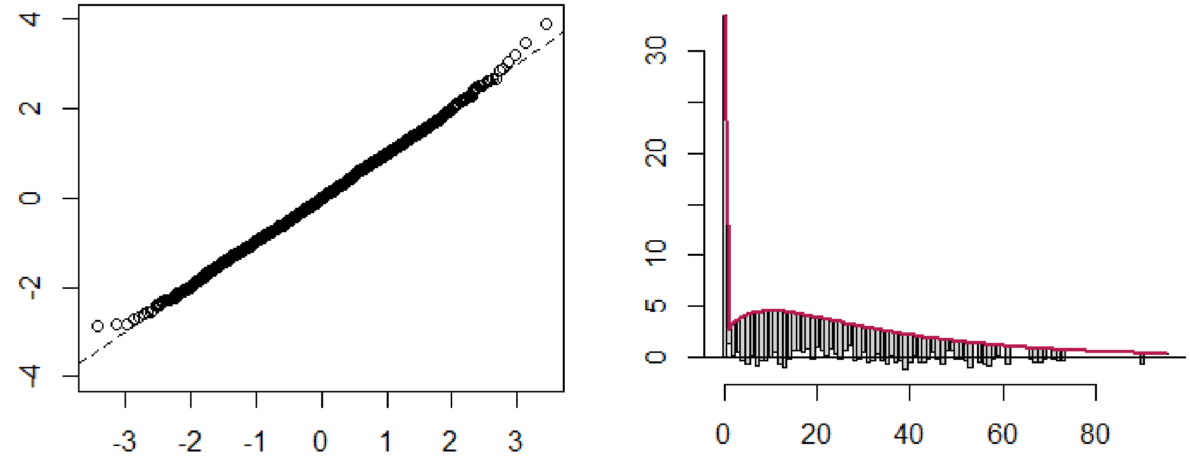

Supplementary Material 5: Summary statistics of variables across sampling plots.

We analyzed a total of 1,720 plots for the occurrence and density model and 843 samples for the persistent, lost and new burrow models. For the remaining plots, no suitable images were available.

A. Summary statistics for continuous variables

Statistic	Mean	St. Dev.	Min	Max
No. burrows	18.28	16.29	0	90
bio11	-14.78	-5.41	-15.9	-13.1
bio17	41.58	2.82	33.0	48.9
d_farm	21338.8	18052.9	260.9	100562.0
d_river	7821.1	8596.6	14.7	420892.0
ndvi_may	118.8	24.9	0.0	199.0
slope	0.6	0.4	0.0	5.3

B. Summary statistics for categorical variables

**Plot distribution per image blocks**	**Observations**
Central	582
Northern	558
Southern	580
**Time period**	
Contemporary	863
Historical	857
**Dominant land use**	
Cropland (overall)	878
Grassland (overall)	751
Other	91
**Soil type**	
Clay and heavy loam	910
Loam	699
Other	111

Supplementary Material 6: Regression results, main model

Regression coefficients, standard errors and significance levels for the occurrence and density model. The first column indicates values for the zero component of the model (predicting non-occurrence of burrows) and the second column shows values for the count component of the model (marmot burrow density). For all the results presented in the paper the predicted probability of non-occurrence was translated into occurrence probability.

Regression coefficients for main model.

	**Estimate (zero comp.)**	**Estimate (count comp.)**
(intercept)	1.17 (0.46) *	2.65 (0.06) ***
period (historical)	-0.79 (0.26) **	0.37 (0.07) ***
dominant land use (grassland)	1.15 (0.49) *	0.25 (0.09) **
dominant land use (other)	2.61 (1.63)	-1.79 (1.06) .
distance to farm		0.14 (0.03) ***
NDVI May		-0.15 (0.03) ***
soil (loam)	2.65 (0.58) ***	
soil (other)	5.46 (1.07) ***	
winter temperature	1.24 (0.31) ***	
precipitation	-1.65 (0.3) ***	
distance to river	0.76 (0.29) *	
slope	-0.01 (0.24)	
period (hist) * land use (grassland)	1 (0.44) *	-0.38 (0.1) ***
period (hist) * land use (other)	1.12 (1.54)	1.44 (1.14)
Random effects		
plot:zone (N=1720)	plot (N=895), zone (N=3)	

Note: Std. errors are given in brackets

*p-value: <0.001 ***, <0.01 **, < 0.05*, <0.1 .*

Supplementary Material 7: Regression results, philopatry models

Regression coefficients, standard errors and significance levels for three models: lost burrows, persistent burrows and new burrows. For each model, the first column indicates the values for the zero component of the model (predicting non-occurrence) and the second column shows values for the count component of the model (number of burrows/ plot)

	**Lost burrows estimates**		**Persistent burrows estimates**		**New burrows estimates**	
	Zero component	Count component	Zero component	Count component	Zero component	Count component
(intercept)	7.9 (2.44) **	1.98 (0.13) ***	2.32 (0.23) ***	1.34 (0.14) ***	1.81 (0.49) ***	1.99 (0.22) ***
no. historical burrows	-2.08 (0.78) **	1.98 (0.13) ***	-0.13 (0.01) ***	0.03 (0) ***	-3.31 (1.8) .	-0.006 (0.004)
cropland-grassland	-0.2 (1.82)	0.02 (0.002) ***	-0.39 (0.4)	0.21 (0.11) .	0.18 (0.63)	0.34 (0.21)
stable grassland	0.12 (1.43)	-0.09 (0.14)	-0.42 (0.27)	0.23 (0.1) *	0.58 (0.46)	0.39 (0.15) *
other land change	-0.85 (1.56)	-0.43 (0.13) ***	1.52 (0.77) .	-0.69 (0.49)	0.79 (0.65)	-0.41 (0.57)
distance to farm		0.07 (0.25)		0.02 (0.04)		-0.06 (0.07)
NDVI May		-0.02 (0.04)		-0.16 (0.04) ***		-0.16 (0.07) *
soil (loam)	-0.94 (1.14)	0.11 (0.05) *	0.07 (0.24)		-0.21 (0.35)	
soil (other)	-2.07 (1.7)		1.43 (0.75) .		1.07 (0.83)	
winter temperature	2.16 (0.98) *		0.18 (0.14)		-0.03 (0.24)	
precipitation	-0.65 (0.66)		-0.52 (0.14) ***		-0.59 (0.23) *	
distance to river	1.01 (1.01)		0.28 (0.16) .		0.25 (0.2)	
slope	-0.18 (0.57)		0 (0.11)		0.29 (0.22)	
Random effect						
zone (N=863)	Variance: 4.2e-06<br>St. dev. 0.002	Variance: 0.02<br>St. dev. 0.14	Variance: 1.4e-08<br>St. dev. 0.0001	Variance: 0.03<br>St. dev. 0.17	Variance: 0.36<br>St. dev. 0.60	Variance: 0.07<br>St. dev. 0.28

Note: Std. errors are given in brackets

*p-value: <0.001 ***, <0.01 **, < 0.05*, <0.1 .*

We estimated the number of burrows lost, persistent and new across land change classes, for a plot that started out with 19 burrows in the historical time period, because the average number of burrows per plot was aprox. 19 in the historical period (in plots where burrows were present). For each of the lost, persistent and new burrow models, regression coefficients, were transformed in probability of occurrence (A), predicted numbers (conditional on the probability of occurrence, count part of the model) (B) and the mean expected number of burrows (C). The results indicate that a plot that started out with 19 burrows in the historical time period lost on average 11.8 burrows in persistent croplands and 7.6 burrows in persistent grasslands. On average, 6.4 burrows persisted in croplands and 4.2 in grasslands.

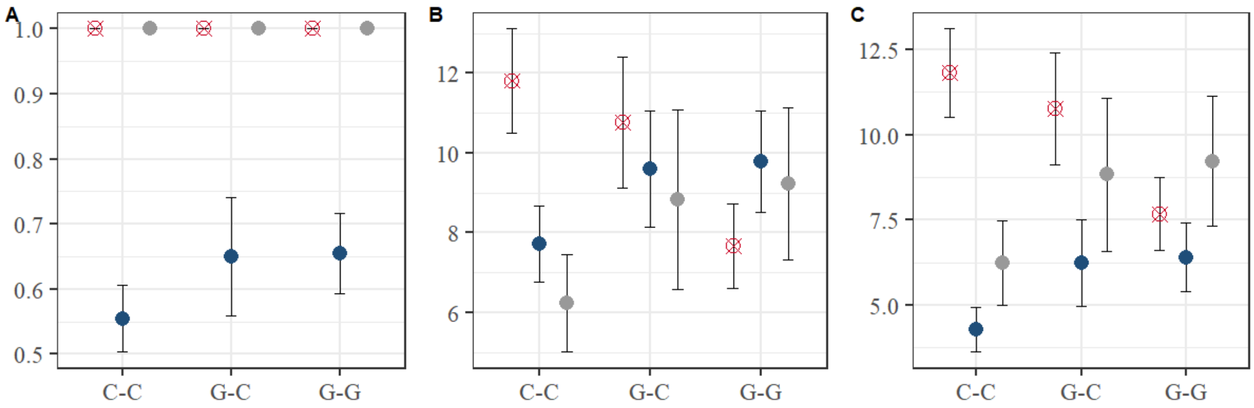

We used the same model to estimate the change in predicted number of burrows lost (A) persistent (B) and new (C), across the possible range of values for the initial number of historical burrows. We found that the number of lost burrows is consistently higher in persistent croplands compared to persistent grasslands (red line), while the number of persistent and new burrows is consistently higher in persistent grasslands (blue line) compared to persistent croplands.

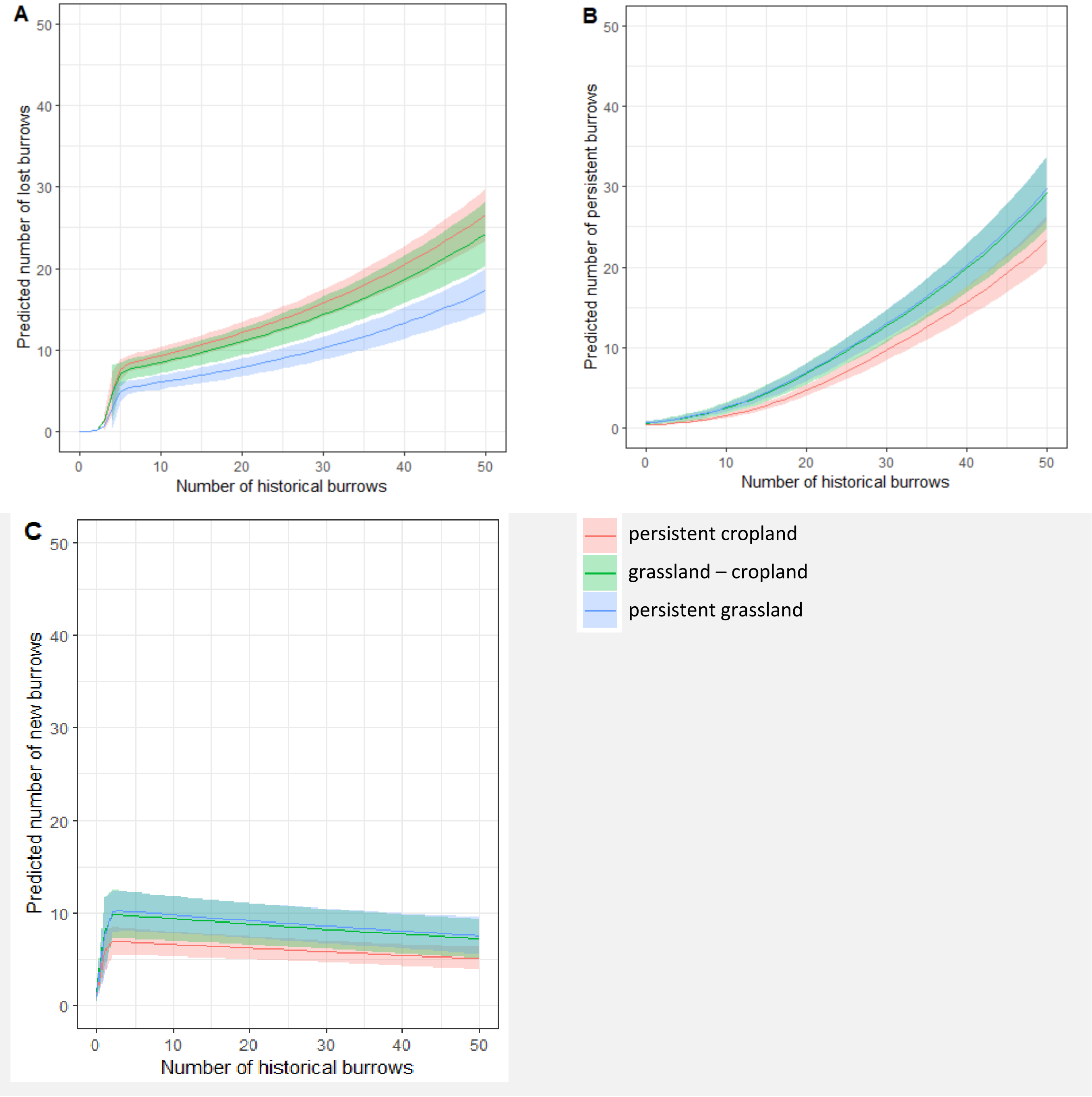

Supplementary Material 8: Active and abandoned cropland

A). Historical and contemporary land-use distribution across the sample plots, including those for which we could separate active and abandoned agriculture. B). For a subset of cropland plots (N=165), we could distinguish between active and abandoned cropland in both time periods. For these plots, we compared the change in burrow density between the historical and the contemporary time period between (a) cropland that was actively cropped in both periods (N=127), and (b) plots that were historically cropped, but abandoned in the contemporary period (N=38). We found no statistical difference in burrow density between these two classes (p=0.354).

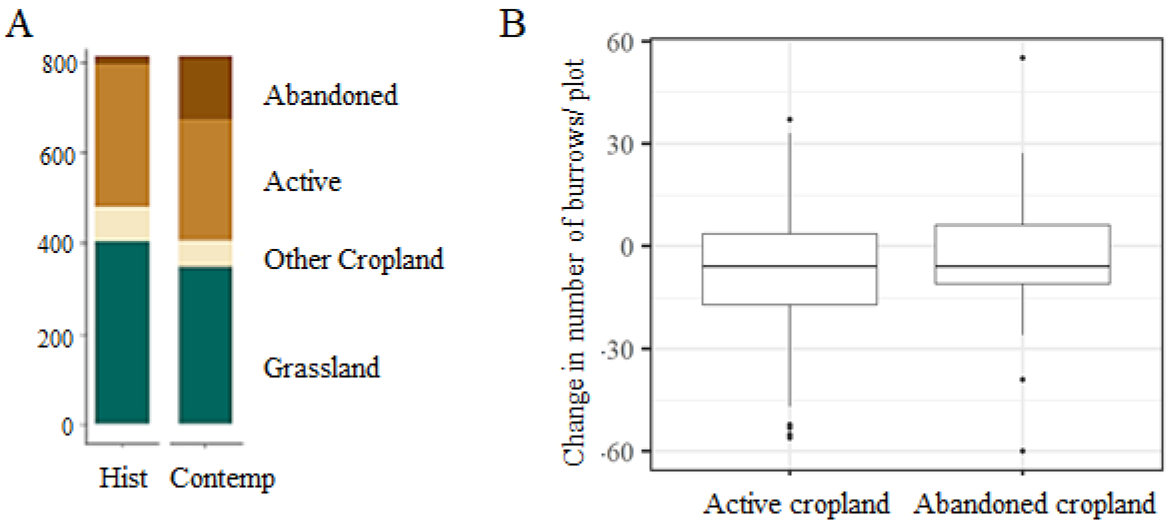

Supplementary Material 9: Virgin Lands Campaign

Data on the extent of agricultural expansion during the Virgin Lands Campaign as well as the pre-campaign was available for N=111 plots classified as cropland in the historical time period. The cropland expansion data was obtained from 1:3,000,000 archival map of the Virgin Lands Campaign from 1953 to 1963 [12,13]. The data covered large parts of the northern two geographical zones in our study, but no data was available for the Qaragandy area.

Extent of the Virgin Lands Campaign

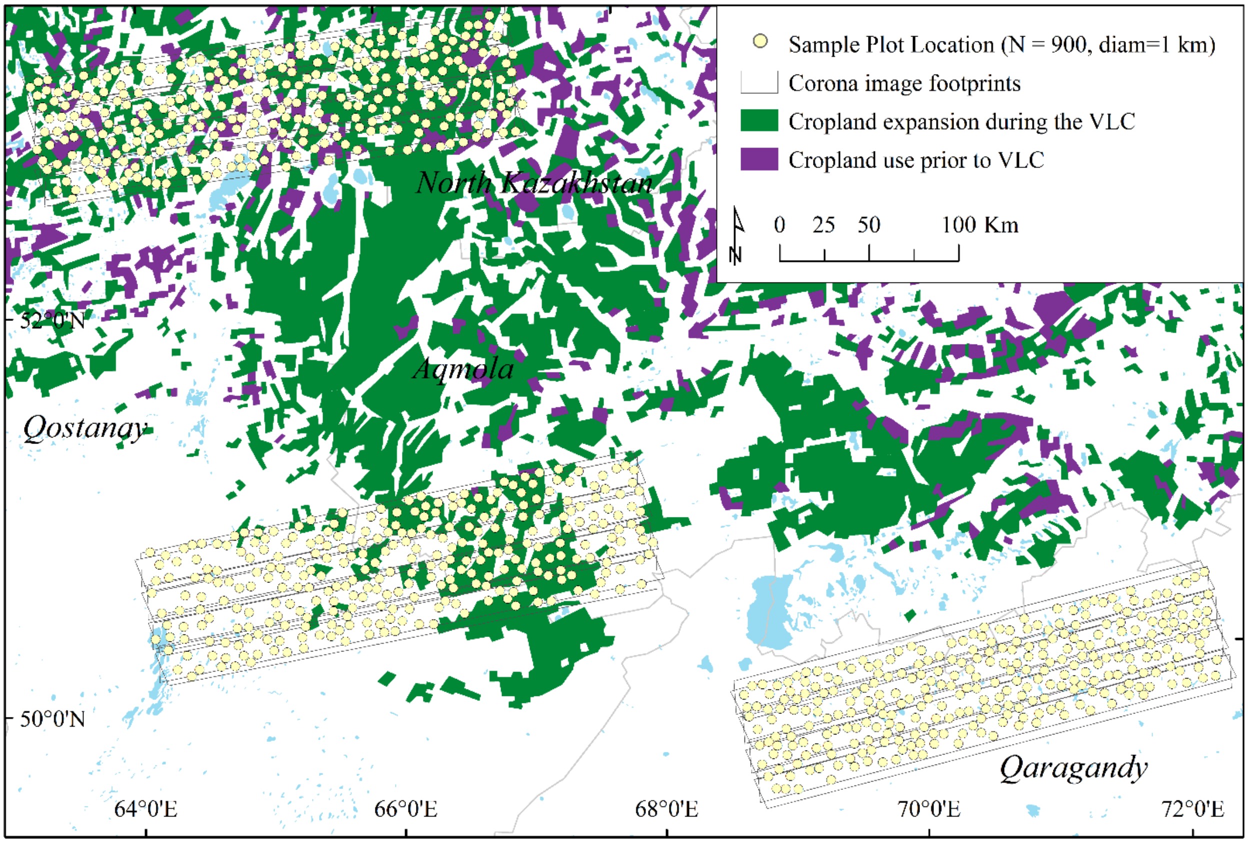

For these 111 plots, we compared the average number of burrows between the historical and contemporary time period for (a) plots that were already used as agriculture before the Virgin Lands Campaign (preVLC) and (b) plots that were converted to cropland during the VLC. We found a steeper decline in average burrow densities in plots cropped already prior to the Virgin Lands Campaign (N=16) compared to plots converted during the Virgin Lands Campaign (N=95), in other words, in areas that had longer agricultural histories. We relied on analysis of covariance (ANCOVA) and Tukeys test of means to check for differences in the average number

of burrows between the two time periods, while considering if the plots were cropped or not prior to the Virgin Lands Campaign (p-value: 0.0006). The results indicated that plots that were farmed prior to the Virgin Lands Campaign (preVLC) lost 22.6 burrows (p-value: 0.002, 95% CI: 6.2-29.3), compared to loosing on average 4 burrows (p-value: 0.388, 95% CI: -2.55-10.61) for VLC plots. These results suggest that burrow declines were steeper on plots cropped for longer time. Due to the only partial overlap with our data, and to potential inconsistencies induced by data sources with vastly different resolution, we did not include this information in further analyses.

Average number of burrows in plots used as agriculture before the Virgin Lands Campaign (preVLC) and converted to cropland during the Virgin Lands Campaign

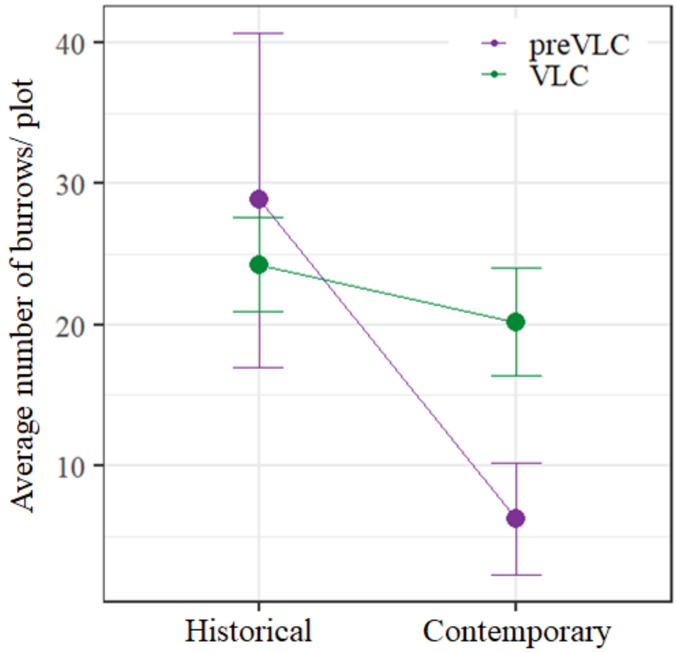

Supplementary Material 10: Recent dynamics

For a subset of plots (N=138), land use and burrow density data were available for more than one point in time between 2000 and 2019. These plots were all classified as cropland (either active or abandoned) in the contemporary period and had marmot burrows present.

Using a general linear model, with plotID as a random effect, we predicted a small increase in burrows numbers between 2000-2019, but the year effect was not significant (p=0.215) (A). When averaging the number of burrows/ plot for each 5-year time-interval between 2000 and 2019 we found small declines following 2005, but this may be related to the small sample size of this group (N=36) compared to the rest (N range: 64-119) (B).

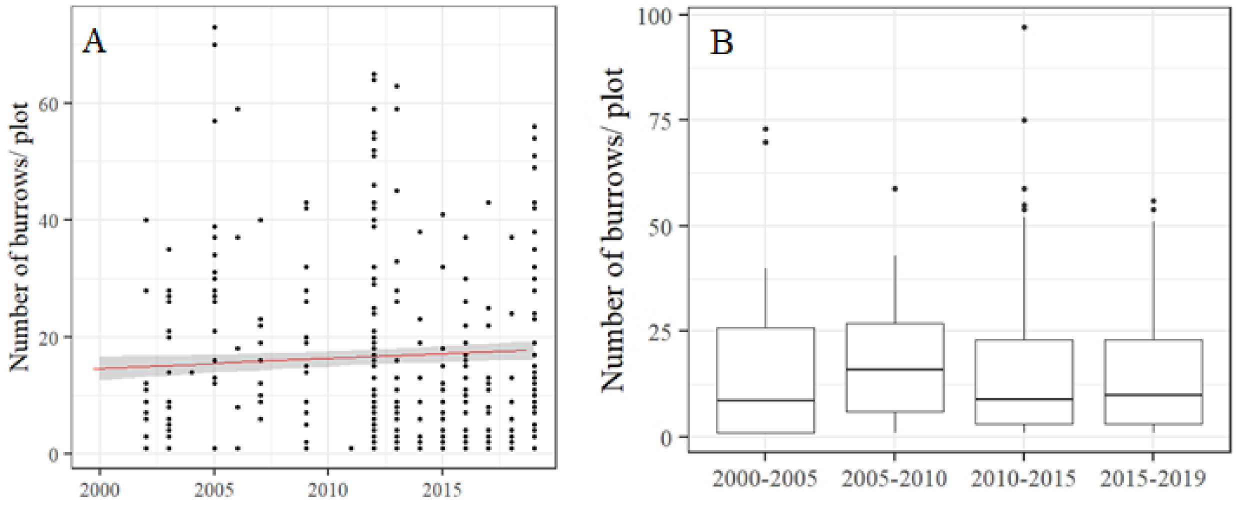

Supplementary Material 11: Interpretation of model results

To predict probability of occurrence and expected number of burrows, we fixed all variables to their mean except the variables *land use* and *time period*. We estimated the random effect of the plot and the zone at the population average (assumed to be zero). In other words, we substituted the assumed population mean for an unknown random effect (also called marginal prediction). We used the package '*glmmTMB*' in R to fit the model and predict values to a new dataset [11,12]. The models used consisted of two parts: the zero component of the model yields regression coefficients on the probability of additional zeros. Coefficients were modelled using a logit link and their interpretation is analogue to a logit model (i.e. by calculating the exponential of the model coefficients), but reflecting the chance of observing additional zeros rather than the probability of occurrence. The probability of occurrence can be calculated from the zero-inflated density using the formula:

$$P(y_i > 0) = (1 - \pi_i) * (1 - (\delta_i/(\mu + \delta_i))\hat{}\delta_i)$$

We show these results in Figure 3A and Figure 4B.

The count part of the model is represented by a negative binomial distribution with parameters μ and δ. These do not have a direct interpretation. However, we can compute the expected number of burrows, provided that burrows are present (i.e. conditional that all additional zeros have been removed) from it. Figure 3B and 4C depict the value E(yi |yi>0), which is

$$E(y_i|y_i > 0) = \mu_i/(1 - (\delta_i/(\mu_i + \delta_i))\hat{}\delta_i)$$

Last but not least, Figure 3C and Figure 4D represent the expected number of burrows per plot, considering the probability of occurrence. Values presented in Figure 3C and Figure 4D are calculated by the formula:

$$E(y_i) = (1 - \pi_i) * \mu_i$$

Where

πi = probability of structural zeros, expected values of zero inflated part of the model

μi = location parameter of the negative binomial part of the model

δi = overdispersion parameter of the negative binomial distribution"

References

1. National Reconnnaissance Office. 2010 The CORONA Story.

2. Song DX, Huang C, Sexton JO, Channan S, Feng M, Townshend JR. 2014 Use of landsat and corona data for mapping forest cover change from the mid-1960s to 2000s: Case studies from the Eastern United States and central Brazil. *ISPRS J. Photogramm. Remote Sens.* **103**, 81–92. (doi:10.1016/j.isprsjprs.2014.09.005)

3. Nita MD, Munteanu C, Gutman G, Abrudan IV, Radeloff VC. 2018 Widespread forest cutting in the aftermath of World War II captured by broad-scale historical Corona spy satellite photography. *Remote Sens. Environ.* **204**, 322–332. (doi:10.1016/j.rse.2017.10.021)

4. Rigina O. 2003 Detection of boreal forest decline with high-resolution panchromatic satellite imagery. *Int. J. Remote Sens.* **24**, 1895–1912. (doi:10.1080/01431160210154894)

5. Maurer JM, Schaefer JM, Rupper S, Corley A. 2019 Acceleration of ice loss across the Himalayas over the past 40 years. *Sci. Adv.* **5**, eaav7266. (doi:10.1126/sciadv.aav7266)

6. Hijmans RJ, Cameron SE, Parra JL, Jones PG, Jarvis A. 2005 Very high resolution interpolated climate surfaces for global land areas. *Int. J. Climatol.* **25**, 1965–1978. (doi:10.1002/joc.1276)

7. Beznosov A, Uspanov U. 1960 *Soils of KazSSR*. (in Russia. Academy of Science KazUSSR.

8. Dara A, Baumann M, Freitag M, Hölzel N, Hostert P, Kamp J, Müller D, Prishchepov A V., Kuemmerle T. 2020 Annual Landsat time series reveal post-Soviet changes in grazing pressure. *Remote Sens. Environ.* **239**, 111667. (doi:10.1016/j.rse.2020.111667)

9. Jarvis A, Reuter HI, Nelson A, Guevara E. In press. Hole-filled SRTM for the globe Version 4, available from the CGIAR-CSI SRTM 90m Database.

10. VTU GSh. 1989 Military 1:100,000 topographic maps. *Mil. Dep. Gen. Staff USSR*

11. WWF/UNEP/GEF. 2006 Econet Central Asia, Project Documents. See https://wwf.ru/en/regions/central-asia/karty.

12. Moscow State University. 1964 Atlas of the Virgin LandsTerritory (Atlas Celinnogo Kraja).

13. Prishchepov AV, Petrick M, Müller D, Schierhorn F, Kraemer R, Kurganova I, Kopsidis M. 2015 Sixty years of the Virgin Lands Campaign in Russia and Kazakhstan: An assessment from an economic, ecological and political perspective. *IAMO Yearb. 201539–55.* , 39–55.
